# Crystal structure of (1,4-diphenyl-4*H*-1,2,4-triazol-3-yl)phenyl­amine di­fluoro­phosphate, and a survey of the di­fluoro­phosphate anion (PO_2_F_2_^−^)

**DOI:** 10.1107/S2056989020006933

**Published:** 2020-06-02

**Authors:** Matthias Weil, Markus Fürst

**Affiliations:** a Institute for Chemical Technologies and Analytics, Division of Structural Chemistry, TU Wien, Getreidemarkt 9/164-SC, A-1060 Vienna, Austria

**Keywords:** crystal structure, Busch’s reagent, PO_2_F_2_^−^ anion, hydrogen bonding, NHC carbene

## Abstract

Nitron is the trivial name of (1,4-diphenyl-4*H*-1,2,4-triazol-3-yl)phenyl­amine (C_20_H_16_N_4_), a triazole derivative used as an analytical reagent for gravimetric analysis of the nitrate anion. The crystal structure of the di­fluoro­phosphate salt of Nitron, 3-anilino-1,4-diphenyl-1*H*-1,2,4-triazol-4-ium di­fluoro­phosphate, C_20_H_17_N_4_^+^·PO_2_F_2_^−^, is reported here. Within the protonated Nitron mol­ecule, the triazole ring, one of the phenyl rings and the NHPh moiety are virtually co-planar, with the third phenyl ring inclined to the least-squares plane of the other three rings by 56.07 (3)°. Inter­molecular N—H⋯O and C—H⋯O hydrogen bonds between cations and di­fluoro­phosphate anions lead to the formation of a three-dimensional network that is consolidated by additional π–π stacking inter­actions between the triazole ring and one of the phenyl rings. Database surveys on inorganic, metal–organic and organic structures comprising the tetra­hedral PO_2_F_2_ group reveal mean bond lengths of P—O = 1.459 (27) Å, P—F = 1.530 (21) Å, and angles of O—P—O = 121.2 (2.9)°, O—P—F = 108.7 (6)°, and F—P—F = 98.5 (2.6)°, using a dataset of 67 independent PO_2_F_2_^−^ anions or PO_2_F_2_ entities.

## Abstract 2

The title compound represents the di­fluoro­phosphate salt of Nitron, an analytical reagent used for gravimetrical analysis of nitrate ions. An analysis of the bond–length distribution and average bond lengths in PO_2_F_2_ tetra­hedra is given.

## Chemical context   

**Figure 1:**
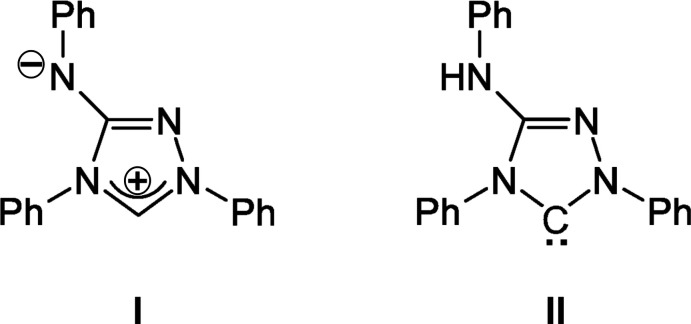
Structure of zwitterionic Nitron (**I**), and of its NHC-carbene tautomer (**II**).

Nitron is the trivial name for the triazole derivative (1,4-diphenyl-4*H*-1,2,4-triazol-3-yl)phenyl­amine, C_20_H_16_N_4_, that shows tautomerism and can be present in its zwitterionic form (**I**) or its NHC-type carbenic form (**II**) (Fig. 1 ▸). Nitron has been utilized as a reagent for gravimetric analysis of the nitrate anion (‘Busch’s reagent’; Busch, 1905 ▸) from slightly acidic solutions under formation of the salt C_20_H_17_N_4_^+^·NO_3_^−^. In recent years, inexpensive Nitron was rediscovered as a stable *N*-heterocyclic carbene (Färber *et al.*, 2012 ▸) that can be reacted with several coinage or other noble metals to yield corresponding metal complexes (Hitzel *et al.*, 2014 ▸; Thie *et al.*, 2016 ▸). The Nitron salt of di­fluoro­phospho­ric acid, C_20_H_17_N_4_^+^·PO_2_F_2_^−^ (**III**) was reported by Lange more than 90 years ago (Lange, 1929 ▸). It can be used as a precursor for obtaining di­fluoro­phosphates of several metals or other cations through metathesis reactions.
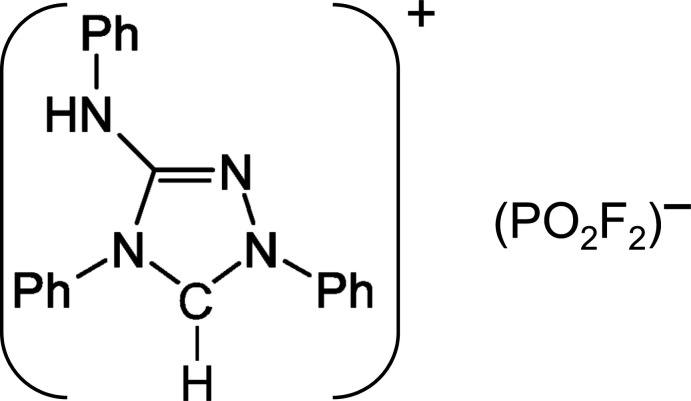


The synthesis, crystallization and structure analysis of **III** are reported here, together with a survey of the structural characteristics of the di­fluoro­phosphate anion present in inorganic, metal–organic and organic compounds.

## Structural commentary   

**Figure 2:**
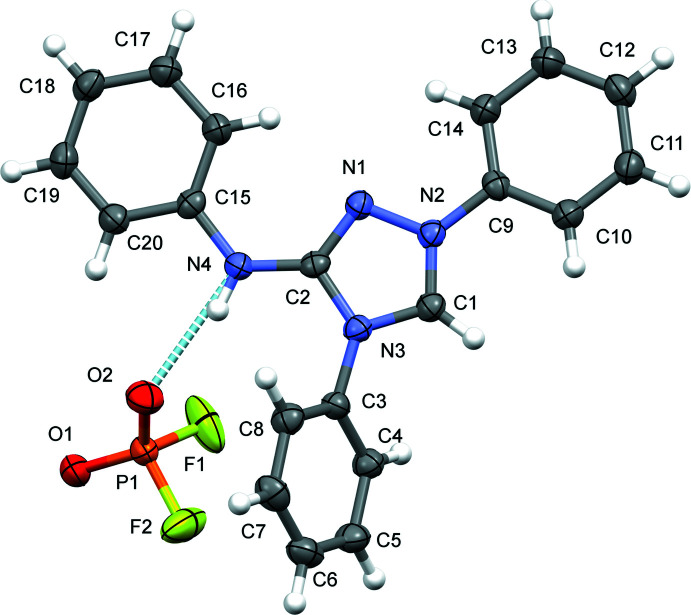
The asymmetric unit of **III**, showing the mol­ecular components with displacement ellipsoids for non-H atoms drawn at the 75% probability level. H atoms are given as spheres of arbitrary radius; N—H⋯O hydrogen bonding between the organic cation and the inorganic anion is shown as a light-blue dashed line.

The asymmetric unit of **III** is composed of a Nitron mol­ecule protonated at the C1 atom of the triazole ring, assuming the NHC-type tautomer **II** to be prevalent in Nitron itself, and a PO_2_F_2_^−^ anion (Fig. 2 ▸).

The central triazole ring (C1, C2, N1–N3), the phenyl ring attached to N2 (C9–C14) and the NHPh moiety attached to C2 (N4, C15–C20) are virtually co-planar with the r.m.s. deviation of the 18 non-H atoms being 0.0666 Å [greatest deviation 0.1250 (13) Å for the phenyl C19 atom]. The third phenyl ring (C3–C8) is inclined to the least-squares plane of the three aforementioned rings by 56.07 (3)° (Fig. 2 ▸). A weak intra­molecular hydrogen bond between a phenyl C—H group (C16—H16) and the free N atom (N1) of the triazole cycle stabilizes the conformation of the mol­ecule (Table 1 ▸).

In **III**, the tetra­hedral di­fluoro­phosphate anion shows the characteristic bond lengths distribution (Table 2 ▸) between two shorter P—O bonds (mean 1.468 Å) and two considerably longer P—F bonds (mean 1.554 Å). The distortion of the anion is evident not only by the two pairs of different bond lengths but even more so by the bond angles that partly deviate considerably from the ideal value of 109.47°. Whereas the O1—P—O2 angle is enlarged by about 14° relative to the ideal value, the F1—P—F2 angle is reduced by about 12°; the four O—P—F angles are rather similar, with a mean of 108.3°.

## Supra­molecular features   

**Figure 3:**
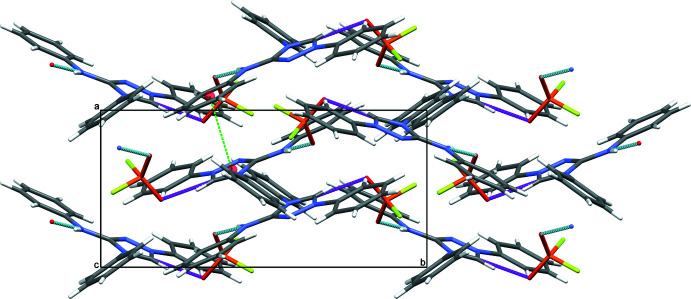
The packing of the organic cations and the inorganic anions in the crystal structure of **III** in a view along [001]. Inter­molecular N—H⋯O and C—H⋯O bonds are shown as light-blue and magenta dashed lines, and π–π stacking inter­actions by green dashed lines.

Aside from Coulombic inter­actions, the cation is hydrogen-bonded by an N—H⋯O inter­action of medium strength between the amino group (N4) of the NHPh moiety and one of the O atoms (O2) of the di­fluoro­phosphate anion. The other O atom (O1) of the anion is the acceptor atom of a weak C—H⋯O hydrogen bond with the protonated carbene C1 atom as the donor group. F atoms are not involved in hydrogen bonding, as frequently observed for related compounds containing the mono­fluoro­phosphate anion PO_3_F^2–^ (Weil *et al.*, 2015 ▸). The two types of hydrogen-bonding inter­actions link the cations and anions into a three-dimensional network structure. Additional π–π stacking between the triazole ring (*Cg*1) and the phenyl ring C15–C20 (*Cg*2) with a centroid-to-centroid distance of *Cg*1⋯*Cg*2(2 − *x*, 1 − *y*, 1 − *z*) = 3.5378 (9) Å and a slippage of 0.643 Å consolidates the packing (Fig. 3 ▸).

## Database survey   

A search of the Cambridge Structural Database (CSD; Version 5.41, last update November 2019; Groom *et al.*, 2016 ▸) for Nitron revealed 17 hits, including various coinage metal complexes of Nitron (EJEZOK, EJICEH, EJICOR, EJIPOE, EJIPUK, EJIQAR, EJIQEV, EJIXOM; Thie *et al.*, 2016 ▸), with selenium bonded to the carbene C atom (EJICIL; Thie *et al.*, 2016 ▸), rhodium complexes (NITLAF, NITLUZ, SAKNAV, SAKNEZ; Hitzel *et al.*, 2014 ▸, Färber *et al.*, 2012 ▸), with a carbodi­thio­ate group attached (SAKNID; Färber *et al.*, 2012 ▸), and isotypic ethyl­enedi­amino­tetra-acetato­aluminate and -gallate complexes (FADJIE, FADJUQ; Ilyukhin & Petrosyant, 2001 ▸). The structure of the hydro­chloride methanol solvate of Nitron (NITLEJ; Hitzel *et al.*, 2014 ▸) is the most similar in comparison to **I** because it shows no direct coordination to a metal and is not derivatized. In (Nitron)^+^Cl^−^· CH_3_OH, the central triazole ring is co-planar with only one phenyl ring (attached to N2). The second phenyl ring (attached to N3) and the NHPh moiety (attached to C2) are inclined to the mean plane by 48.13 (7) and 31.42 (6)°, respectively. The chloride anion is hydrogen-bonded through N—H⋯Cl and O—H⋯Cl inter­actions to the protonated Nitron mol­ecule and the methanol solvent mol­ecule, respectively. In all structures comprising Nitron, the N atom (equivalent to N4 in the title structure) is protonated like in **II**.

A search of the Inorganic Crystal Structure Database (ICSD; Zagorac *et al.*, 2019 ▸) and the CSD for the di­fluoro­phosphate anion or the PO_2_F_2_ entity revealed the crystal structures of twelve inorganic and 30 metal–organic or organic compounds (Table 3 ▸). For a statistical analysis of bond lengths and angles within a PO_2_F_2_ tetra­hedron, only ordered PO_2_F_2_ groups were considered. In summary, 67 independent PO_2_F_2_ tetra­hedra were used, leading to the following averaged bond lengths and angles: P—O = 1.459 (27) Å, P—F = 1.530 (21) Å; O—P—O = 121.2 (2.9)°, O—P—F = 108.7 (6)°, F—P—F = 98.5 (2.6)°. It is evident that the bond lengths and angles observed in **III** (Table 2 ▸) fall within these limits.

## Synthesis and crystallization   

In a nickel crucible, P_2_O_5_ (2.67 g) and NH_4_F (1.86 g) were thoroughly mixed. The open crucible was placed on a hot plate (≃ 420 K) where a vehement reaction took place within a few seconds. The crucible was then taken from the plate and cooled to room temperature. The obtained solid was dissolved in 50 ml water to which ammonia solution (25%_wt_) was added until neutralisation. Subsequently, the pH was adjusted to *ca*. 5 with a few drops of glacial acetic acid. Nitron (3 g) was then added in small portions to the cooled (273 K) acetic solution under constant stirring for about two h. The formed solid was separated by suction filtration and recrystallized from diluted acetic acid solution. Storing the solution in a refrigerator at 280 K overnight resulted in the formation of light-brown crystals of the title compound with a rod-like form; yield 60%.

## Refinement   

Crystal data, data collection and structure refinement details are summarized in Table 4 ▸. The H atom attached to N1 was discernible in a difference-Fourier map and was refined freely.

## Supplementary Material

Crystal structure: contains datablock(s) I. DOI: 10.1107/S2056989020006933/pk2629sup1.cif

Structure factors: contains datablock(s) I. DOI: 10.1107/S2056989020006933/pk2629Isup2.hkl

Supplementary material: Averaged bond lengths and angles in PO2F2 anions or PO2F2 entities present in metal-organic and organic compounds. DOI: 10.1107/S2056989020006933/pk2629sup3.pdf

CCDC reference: 2005191

Additional supporting information:  crystallographic information; 3D view; checkCIF report

## Figures and Tables

**Table 1:** Hydrogen-bond geometry (Å, °)

*D*—H⋯*A*	*D*—H	H⋯*A*	*D*⋯*A*	*D*—H⋯*A*
C1—H1⋯O1^i^	0.95	2.02	2.9576 (17)	168
C20—H20⋯O2	0.95	2.44	3.2291 (17)	140
C16—H16⋯N1	0.95	2.22	2.8700 (18)	124
C10—H10⋯O1^i^	0.95	2.45	3.3673 (17)	161
N4—H1*N*⋯O2	0.857 (19)	2.025 (19)	2.8614 (16)	165.0 (17)

Symmetry code: (i) 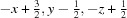.

**Table 2:** Selected geometric parameters (Å, °)

P1—O1	1.4684 (11)	P1—F2	1.5510 (10)
P1—O2	1.4686 (11)	P1—F1	1.5568 (10)
			
O1—P1—O2	123.22 (6)	O1—P1—F1	108.21 (6)
O1—P1—F2	107.75 (6)	O2—P1—F1	108.10 (6)
O2—P1—F2	109.20 (6)	F2—P1—F1	97.26 (7)

**Table 3:** Averaged bond lengths (Å) and angles (°) in PO_2_F_2_ tetra­hedra present in several compounds

Compound	Independent PO_2_F_2_ groups	P—O	P—F	O—P—O	O—P—F	F—P—F
NH_4_PO_2_F_2_^*a*^	1	1.457	1.541	118.7	109.5	98.6
KPO_2_F_2_^*b*^	1	1.470	1.575	122.4	108.7	97.1
CsPO_2_F_2_^*c*^	1	1.480	1.545	121.0	108.5	99.0
LiB(PO_2_F_2_)_4_^*d*^	4 (1 disordered)	1.483	1.520	119.7	108.6	100.5
AgPO_2_F_2_^*e*^	3	1.459	1.511	119.9	108.1	103.3
Ag^I^_4_Ag^II^_5_(PO_2_F_2_)_14_^*e*^	7	1.481	1.511	117.8	109.4	99.9
Cu(PO_2_F_2_)_2_^*f*^	3	1.450	1.496	119.1	109.5	97.6
Cs_2_Fe_2_F_3_(PO_3_F)_2_(PO_2_F_2_)^*g*^	1	1.512	1.555	117.8	108.2	106.2
KFe_2_(PO_2_F_2_)(PO_3_F)_2_F_2_^*g*^	1	1.509	1.569	115.6	109.3	103.4
SbF_4_(PO_2_F_2_)^*h*^	1	1.500	1.500	117.4	108.5	104.6
(NH_4_)Mn_3_(PO_3_F)_2_(PO_2_F_2_)F_2_^*i*^	1	1.482	1.572	116.6	109.8	100.0
(NH_4_)Co_3_(PO_3_F)_2_(PO_2_F_2_)F_2_^*i*^	1	1.486	1.554	114.9	110.1	100.8
Organic and metal–organic compounds^*j*^	42	1.449	1.532	122.3	108.5	97.5

(*a*) Harrison & Trotter (1969 ▸); (*b*) Harrison *et al.* (1966 ▸); (*c*) Trotter & Whitlow (1967 ▸); (*d*) Schulz *et al.* (2015 ▸); (*e*) Malinowski *et al.* (2015 ▸); (*f*) Begley *et al.* (1985 ▸); (*g*) Keates *et al.* (2013 ▸); (*h*) Schneider *et al.* (2001 ▸); (*i*) Jiang *et al.* (2020 ▸); (*j*) A detailed list of entries for these compounds is given in Table S1 of the supporting information.

**Table 4:** Experimental details

Crystal data	
Chemical formula	C_20_H_17_N_4_^+^·PO_2_F_2_^−^
*M*_r_	414.35
Crystal system, space group	Monoclinic, *P*2_1_/*n*
Temperature (K)	100
*a*, *b*, *c* (Å)	7.3811 (5), 14.9963 (9), 16.9217 (10)
β (°)	102.138 (2)
*V* (Å^3^)	1831.2 (2)
*Z*	4
Radiation type	Mo *K*α
μ (mm^−1^)	0.20
Crystal size (mm)	0.50 × 0.10 × 0.10

Data collection	
Diffractometer	Bruker APEXII CCD
Absorption correction	Multi-scan (*SADABS*; Krause *et al.*, 2015 ▸)
*T*_min_, *T*_max_	0.701, 0.746
No. of measured, independent and observed [*I* > 2σ(*I*)] reflections	30253, 5325, 4077
*R*_int_	0.046
(sin θ/λ)_max_ (Å^−1^)	0.703

Refinement	
*R*[*F* ^2^ > 2σ(*F* ^2^)], *wR*(*F* ^2^), *S*	0.039, 0.100, 1.03
No. of reflections	5325
No. of parameters	266
H-atom treatment	H atoms treated by a mixture of independent and constrained refinement
Δρ_max_, Δρ_min_ (e Å^−3^)	0.38, −0.36

Computer programs: *APEX2* and *SAINT* (Bruker, 2016 ▸), *SHELXT* (Sheldrick, 2015*a* ▸), *SHELXL* (Sheldrick, 2015*b* ▸), *Mercury* (Macrae *et al.*, 2020 ▸) and *publCIF* (Westrip, 2010 ▸).

## supplementary crystallographic information

### Crystal data

**Table:**

C_20_H_17_N_4_^+^·PO_2_F_2_^−^	*F*(000) = 856
*M**_r_* = 414.35	*D*_x_ = 1.503 Mg m^−^^3^
Monoclinic, *P*2_1_/*n*	Mo *K*α radiation, λ = 0.71073 Å
*a* = 7.3811 (5) Å	Cell parameters from 7088 reflections
*b* = 14.9963 (9) Å	θ = 2.5–29.9°
*c* = 16.9217 (10) Å	µ = 0.20 mm^−^^1^
β = 102.138 (2)°	*T* = 100 K
*V* = 1831.2 (2) Å^3^	Bar, light-brown
*Z* = 4	0.50 × 0.10 × 0.10 mm

### Data collection

**Table:**

Bruker APEXII CCD diffractometer	4077 reflections with *I* > 2σ(*I*)
ω– and f–scans	*R*_int_ = 0.046
Absorption correction: multi-scan (*SADABS*; Krause *et al.*, 2015)	θ_max_ = 30.0°, θ_min_ = 2.5°
*T*_min_ = 0.701, *T*_max_ = 0.746	*h* = −10→10
30253 measured reflections	*k* = −21→21
5325 independent reflections	*l* = −23→23

### Refinement

**Table:**

Refinement on *F*^2^	0 restraints
Least-squares matrix: full	Hydrogen site location: mixed
*R*[*F*^2^ > 2σ(*F*^2^)] = 0.039	H atoms treated by a mixture of independent and constrained refinement
*wR*(*F*^2^) = 0.100	*w* = 1/[σ^2^(*F*_o_^2^) + (0.0405*P*)^2^ + 0.7893*P*] where *P* = (*F*_o_^2^ + 2*F*_c_^2^)/3
*S* = 1.03	(Δ/σ)_max_ = 0.001
5325 reflections	Δρ_max_ = 0.38 e Å^−^^3^
266 parameters	Δρ_min_ = −0.36 e Å^−^^3^

### Special details

**Table:**

Geometry. All esds (except the esd in the dihedral angle between two l.s. planes) are estimated using the full covariance matrix. The cell esds are taken into account individually in the estimation of esds in distances, angles and torsion angles; correlations between esds in cell parameters are only used when they are defined by crystal symmetry. An approximate (isotropic) treatment of cell esds is used for estimating esds involving l.s. planes.

### Fractional atomic coordinates and isotropic or equivalent isotropic displacement parameters (Å^2^)

**Table:**

	*x*	*y*	*z*	*U*_iso_*/*U*_eq_	
P1	0.92663 (5)	0.62394 (2)	0.23419 (2)	0.01781 (9)	
F1	1.02420 (15)	0.53730 (7)	0.27209 (7)	0.0427 (3)	
F2	0.82504 (15)	0.58249 (8)	0.15294 (6)	0.0399 (3)	
O1	1.06913 (14)	0.68482 (7)	0.21683 (6)	0.0215 (2)	
O2	0.78943 (15)	0.65005 (7)	0.28139 (6)	0.0256 (2)	
N1	0.67953 (16)	0.42442 (7)	0.49191 (7)	0.0167 (2)	
N2	0.60128 (16)	0.34410 (7)	0.46219 (7)	0.0159 (2)	
N3	0.59412 (16)	0.42525 (7)	0.35731 (7)	0.0160 (2)	
N4	0.74014 (17)	0.55678 (8)	0.42288 (7)	0.0172 (2)	
C1	0.55145 (19)	0.34448 (9)	0.38303 (8)	0.0173 (3)	
H1	0.495559	0.296572	0.349946	0.021*	
C2	0.67538 (18)	0.47308 (9)	0.42636 (8)	0.0154 (3)	
C3	0.55076 (19)	0.45013 (9)	0.27301 (8)	0.0164 (3)	
C4	0.6100 (2)	0.39458 (9)	0.21783 (8)	0.0192 (3)	
H4	0.686529	0.344484	0.235566	0.023*	
C5	0.5545 (2)	0.41403 (10)	0.13613 (8)	0.0223 (3)	
H5	0.592902	0.376766	0.097312	0.027*	
C6	0.4434 (2)	0.48749 (10)	0.11080 (9)	0.0234 (3)	
H6	0.405364	0.500038	0.054732	0.028*	
C7	0.3878 (2)	0.54265 (10)	0.16687 (9)	0.0228 (3)	
H7	0.313089	0.593352	0.149122	0.027*	
C8	0.4405 (2)	0.52423 (9)	0.24886 (8)	0.0201 (3)	
H8	0.402026	0.561559	0.287605	0.024*	
C9	0.58060 (18)	0.27329 (9)	0.51670 (8)	0.0161 (3)	
C10	0.4911 (2)	0.19539 (9)	0.48580 (8)	0.0199 (3)	
H10	0.445350	0.188499	0.429318	0.024*	
C11	0.4701 (2)	0.12795 (10)	0.53908 (9)	0.0221 (3)	
H11	0.410626	0.073931	0.518867	0.027*	
C12	0.5349 (2)	0.13848 (10)	0.62172 (9)	0.0221 (3)	
H12	0.517537	0.092364	0.657898	0.026*	
C13	0.6250 (2)	0.21647 (10)	0.65123 (9)	0.0242 (3)	
H13	0.670021	0.223552	0.707742	0.029*	
C14	0.6498 (2)	0.28444 (10)	0.59862 (8)	0.0210 (3)	
H14	0.713296	0.337592	0.618605	0.025*	
C15	0.82708 (19)	0.60945 (9)	0.48991 (8)	0.0162 (3)	
C16	0.8404 (2)	0.58403 (9)	0.57031 (8)	0.0200 (3)	
H16	0.792304	0.528252	0.582833	0.024*	
C17	0.9249 (2)	0.64124 (10)	0.63198 (9)	0.0226 (3)	
H17	0.933453	0.623820	0.686619	0.027*	
C18	0.9968 (2)	0.72284 (9)	0.61568 (9)	0.0214 (3)	
H18	1.052684	0.761540	0.658329	0.026*	
C19	0.9854 (2)	0.74688 (10)	0.53521 (9)	0.0211 (3)	
H19	1.036019	0.802151	0.522932	0.025*	
C20	0.9009 (2)	0.69113 (9)	0.47280 (8)	0.0198 (3)	
H20	0.893375	0.708627	0.418243	0.024*	
H1N	0.741 (3)	0.5776 (12)	0.3758 (11)	0.031 (5)*	

### Atomic displacement parameters (Å^2^)

**Table:**

	*U*^11^	*U*^22^	*U*^33^	*U*^12^	*U*^13^	*U*^23^
P1	0.02253 (19)	0.01490 (16)	0.01649 (17)	−0.00094 (14)	0.00522 (14)	0.00142 (13)
F1	0.0456 (6)	0.0255 (5)	0.0631 (7)	0.0121 (5)	0.0255 (6)	0.0221 (5)
F2	0.0414 (6)	0.0531 (7)	0.0280 (5)	−0.0244 (5)	0.0132 (4)	−0.0184 (5)
O1	0.0245 (5)	0.0200 (5)	0.0200 (5)	−0.0034 (4)	0.0045 (4)	0.0023 (4)
O2	0.0291 (6)	0.0270 (6)	0.0231 (5)	0.0005 (5)	0.0110 (5)	0.0005 (4)
N1	0.0190 (6)	0.0140 (5)	0.0175 (5)	−0.0006 (4)	0.0049 (5)	−0.0010 (4)
N2	0.0175 (6)	0.0137 (5)	0.0169 (5)	0.0000 (4)	0.0043 (4)	−0.0015 (4)
N3	0.0184 (6)	0.0155 (5)	0.0144 (5)	0.0009 (4)	0.0040 (4)	−0.0012 (4)
N4	0.0225 (6)	0.0151 (5)	0.0147 (5)	−0.0011 (5)	0.0056 (5)	0.0001 (4)
C1	0.0187 (6)	0.0160 (6)	0.0178 (6)	0.0007 (5)	0.0049 (5)	−0.0010 (5)
C2	0.0151 (6)	0.0168 (6)	0.0148 (6)	0.0020 (5)	0.0044 (5)	−0.0019 (5)
C3	0.0173 (6)	0.0173 (6)	0.0145 (6)	−0.0011 (5)	0.0033 (5)	0.0008 (5)
C4	0.0216 (7)	0.0184 (6)	0.0178 (6)	0.0015 (5)	0.0049 (5)	−0.0010 (5)
C5	0.0253 (7)	0.0255 (7)	0.0170 (6)	−0.0019 (6)	0.0066 (6)	−0.0028 (5)
C6	0.0222 (7)	0.0305 (8)	0.0168 (6)	−0.0036 (6)	0.0025 (6)	0.0034 (6)
C7	0.0198 (7)	0.0235 (7)	0.0239 (7)	0.0033 (6)	0.0020 (6)	0.0063 (6)
C8	0.0203 (7)	0.0195 (7)	0.0208 (7)	0.0020 (5)	0.0054 (6)	−0.0015 (5)
C9	0.0160 (6)	0.0149 (6)	0.0184 (6)	0.0011 (5)	0.0064 (5)	0.0015 (5)
C10	0.0232 (7)	0.0191 (7)	0.0174 (6)	−0.0025 (5)	0.0045 (5)	−0.0016 (5)
C11	0.0242 (7)	0.0184 (7)	0.0243 (7)	−0.0033 (6)	0.0062 (6)	−0.0017 (5)
C12	0.0235 (7)	0.0209 (7)	0.0221 (7)	−0.0018 (6)	0.0057 (6)	0.0052 (5)
C13	0.0289 (8)	0.0243 (7)	0.0184 (7)	−0.0047 (6)	0.0026 (6)	0.0012 (5)
C14	0.0255 (7)	0.0183 (7)	0.0188 (7)	−0.0041 (6)	0.0038 (6)	−0.0014 (5)
C15	0.0152 (6)	0.0157 (6)	0.0179 (6)	0.0022 (5)	0.0037 (5)	−0.0019 (5)
C16	0.0251 (7)	0.0163 (6)	0.0185 (6)	−0.0010 (6)	0.0042 (6)	0.0011 (5)
C17	0.0287 (8)	0.0201 (7)	0.0180 (6)	0.0005 (6)	0.0027 (6)	0.0004 (5)
C18	0.0234 (7)	0.0168 (6)	0.0219 (7)	0.0013 (6)	0.0002 (6)	−0.0034 (5)
C19	0.0225 (7)	0.0149 (6)	0.0256 (7)	−0.0012 (5)	0.0044 (6)	0.0001 (5)
C20	0.0232 (7)	0.0182 (6)	0.0190 (7)	0.0001 (5)	0.0064 (6)	0.0008 (5)

### Geometric parameters (Å, º)

**Table:**

P1—O1	1.4684 (11)	C7—H7	0.9500
P1—O2	1.4686 (11)	C8—H8	0.9500
P1—F2	1.5510 (10)	C9—C14	1.3832 (18)
P1—F1	1.5568 (10)	C9—C10	1.3890 (19)
N1—C2	1.3227 (17)	C10—C11	1.385 (2)
N1—N2	1.3840 (15)	C10—H10	0.9500
N2—C1	1.3124 (17)	C11—C12	1.389 (2)
N2—C9	1.4356 (17)	C11—H11	0.9500
N3—C1	1.3467 (17)	C12—C13	1.386 (2)
N3—C2	1.3944 (16)	C12—H12	0.9500
N3—C3	1.4439 (16)	C13—C14	1.391 (2)
N4—C2	1.3488 (17)	C13—H13	0.9500
N4—C15	1.4200 (17)	C14—H14	0.9500
N4—H1N	0.857 (19)	C15—C20	1.3957 (19)
C1—H1	0.9500	C15—C16	1.3962 (18)
C3—C8	1.3870 (19)	C16—C17	1.3930 (19)
C3—C4	1.3887 (19)	C16—H16	0.9500
C4—C5	1.3877 (19)	C17—C18	1.385 (2)
C4—H4	0.9500	C17—H17	0.9500
C5—C6	1.386 (2)	C18—C19	1.394 (2)
C5—H5	0.9500	C18—H18	0.9500
C6—C7	1.385 (2)	C19—C20	1.3878 (19)
C6—H6	0.9500	C19—H19	0.9500
C7—C8	1.3877 (19)	C20—H20	0.9500
			
O1—P1—O2	123.22 (6)	C3—C8—H8	120.7
O1—P1—F2	107.75 (6)	C7—C8—H8	120.7
O2—P1—F2	109.20 (6)	C14—C9—C10	121.68 (13)
O1—P1—F1	108.21 (6)	C14—C9—N2	119.20 (12)
O2—P1—F1	108.10 (6)	C10—C9—N2	119.12 (12)
F2—P1—F1	97.26 (7)	C11—C10—C9	118.60 (13)
C2—N1—N2	103.95 (10)	C11—C10—H10	120.7
C1—N2—N1	111.91 (11)	C9—C10—H10	120.7
C1—N2—C9	127.89 (12)	C10—C11—C12	120.69 (14)
N1—N2—C9	120.19 (11)	C10—C11—H11	119.7
C1—N3—C2	106.29 (11)	C12—C11—H11	119.7
C1—N3—C3	122.23 (11)	C13—C12—C11	119.78 (13)
C2—N3—C3	131.44 (11)	C13—C12—H12	120.1
C2—N4—C15	125.98 (12)	C11—C12—H12	120.1
C2—N4—H1N	116.8 (12)	C12—C13—C14	120.39 (13)
C15—N4—H1N	116.7 (12)	C12—C13—H13	119.8
N2—C1—N3	107.41 (12)	C14—C13—H13	119.8
N2—C1—H1	126.3	C9—C14—C13	118.84 (13)
N3—C1—H1	126.3	C9—C14—H14	120.6
N1—C2—N4	127.16 (12)	C13—C14—H14	120.6
N1—C2—N3	110.42 (11)	C20—C15—C16	119.42 (12)
N4—C2—N3	122.41 (12)	C20—C15—N4	116.94 (12)
C8—C3—C4	122.04 (13)	C16—C15—N4	123.64 (12)
C8—C3—N3	119.51 (12)	C17—C16—C15	119.38 (13)
C4—C3—N3	118.27 (12)	C17—C16—H16	120.3
C5—C4—C3	118.36 (13)	C15—C16—H16	120.3
C5—C4—H4	120.8	C18—C17—C16	121.67 (13)
C3—C4—H4	120.8	C18—C17—H17	119.2
C6—C5—C4	120.43 (14)	C16—C17—H17	119.2
C6—C5—H5	119.8	C17—C18—C19	118.44 (13)
C4—C5—H5	119.8	C17—C18—H18	120.8
C7—C6—C5	120.29 (13)	C19—C18—H18	120.8
C7—C6—H6	119.9	C20—C19—C18	120.86 (13)
C5—C6—H6	119.9	C20—C19—H19	119.6
C6—C7—C8	120.31 (14)	C18—C19—H19	119.6
C6—C7—H7	119.8	C19—C20—C15	120.22 (13)
C8—C7—H7	119.8	C19—C20—H20	119.9
C3—C8—C7	118.56 (13)	C15—C20—H20	119.9

### Hydrogen-bond geometry (Å, º)

**Table:**

*D*—H···*A*	*D*—H	H···*A*	*D*···*A*	*D*—H···*A*
C1—H1···O1^i^	0.95	2.02	2.9576 (17)	168
C20—H20···O2	0.95	2.44	3.2291 (17)	140
C16—H16···N1	0.95	2.22	2.8700 (18)	124
C10—H10···O1^i^	0.95	2.45	3.3673 (17)	161
N4—H1*N*···O2	0.857 (19)	2.025 (19)	2.8614 (16)	165.0 (17)

Symmetry code: (i) −*x*+3/2, *y*−1/2, −*z*+1/2.
