# Supplementary material for: Crystal structure of (1,4-diphenyl-4H-1,2,4-triazol-3-yl)phenyl­amine di­fluoro­phosphate, and a survey of the di­fluoro­phosphate anion (PO2F2 −)
Source: Acta Crystallogr E Crystallogr Commun. 2020 Jun 2;76(Pt 7):1003–6. doi: 10.1107/S2056989020006933 (PMC7336792; doi:10.1107/S2056989020006933)
Supplement: Supplementary file 3 [file e-76-01003-sup3.pdf]

Supplementary material for submission „Crystal structure of 1,4-diphenyl-4*H*-[1,2,4]triazol-3-yl)-phenylammmonium difluorophosphate (check name) and a survey of the difluorophosphate anion ($PO_2F_2^-$)“

Table S1. Averaged bond lengths (Å) and angles (°) in $PO_2F_2^-$ anions or PO_2F_2 entities present in metal-organic and organic compounds

**CCDC code**	**Independent PO_2F_2 groups**	**P—O**	**P—F**	**O—P—O**	**O—P—F**	**F—P—F**
BASFUX	1	1.441	1.558	123.7	108.4	96.2
BATPOD	2	1.459	1.549	122.7	108.6	96.8
BOXHAY	1	1.457	1.532	123.2	108.5	96.8
BOXHEC	1	1.433	1.543	122.4	108.9	94.9
CAHNEF	3	1.464	1.540	121.8	108.6	98.1
DELYEY	1	1.444	1.511	121.3	109.4	94.6
ETACAP	1	1.411	1.502	125.9	107.9	95.0
FOSDOI	4 (1 disordered)	1.483	1.520	119.7	108.6	100.5
GAJGII	1	1.466	1.556	121.7	109.0	96.0
LAHDUT	2	1.464	1.536	121.2	108.6	98.8
LEBROC	1	1.459	1.541	122.4	108.8	96.0
LEHPUA	2	1.460	1.557	123.2	108.5	96.4
LEWYIL	2 (1 disordered)	1.464	1.539	124.0	107.9	98.2
LEYRUC	2	1.453	1.513	123.2	108.2	97.7
LOLTUC	1	1.4211	1.510	120.8	109.0	97.6
MEFKAJ	2	1.437	1.530	123.1	108.4	97.2
MEFKEN	2	1.448	1.529	121.9	108.7	97.3
POWCOT	2	1.490	1.555	118.7	108.8	101.2
PUCPIM	1	1.466	1.521	118.4	109.0	101.1
QIGFIX	1	1.403	1.510	124.3	108.1	96.5
OGILIZ	1	1.454	1.513	120.0	108.8	99.5
QUDVUH	1	1.346	1.522	131.4	107.1	90.4
QUDWAO	1	1.446	1.529	122.2	108.7	97.1
RIKQAD	1	1.459	1.550	123.9	108.2	97.1
SUSNAX	1	1.429	1.582	123.7	107.8	98.4
TEJYIQ	1	1.459	1.534	121.8	108.7	97.7
WEQWUL	1	1.464	1.528	119.8	109.0	98.7
WIBMID	1	1.450	1.522	125.4	107.9	95.6
XIJWOD	1	1.432	1.522	123.6	108.1	97.6
ZUVAX	1	1.455	1.532	122.8	108.4	97.5
